# Physical activity changes in the winter in older persons living in northern Japan: a prospective study

**DOI:** 10.1186/s12877-015-0034-z

**Published:** 2015-04-10

**Authors:** Atsushi Mizumoto, Hikaru Ihira, Keitaro Makino, Shigeyuki Saitoh, Hirofumi Ohnishi, Taketo Furuna

**Affiliations:** School of Health Sciences, Sapporo Medical University, South 1, West 17, Chuo-ku, Sapporo, Hokkaido 060-8556 Japan; Department of Cardiovascular, Renal and Metabolic Medicine, Sapporo Medical University, Sapporo, Hokkaido Japan; Department of Public Health, Sapporo Medical University, Sapporo, Hokkaido Japan

**Keywords:** Snow, Outdoor excursions, Step count, Moderate-vigorous physical activity, Accelerometer, Season

## Abstract

**Background:**

Physical activity affects physical and mental health, prevents disease, and improves quality of life. However, physical activity also decreases with age in older persons, and is affected by adverse climatic periods. This study examined changes in physical activity during the winter season in older persons (≥75 years of age) who were living in northern Japan, and evaluated the factors that were associated with this decline.

**Methods:**

A total of 201 older persons (83 men and 118 women; mean age, 79.7 ± 3.8 years) participated in two separate tests that were conducted in November 2012 and February 2013. Physical activity was monitored using the Kenz Lifecorder, and mean step counts and moderate-vigorous physical activity (MVPA) times were calculated over a 1-week period. We also measured body mass index, handgrip strength, normal walking speed, functional capacity, exercise habits, snow-shovelling habits, a change in frequency (between early winter and midwinter) of outdoor excursions, the length of outdoor excursions, social support, and morbidity.

**Results:**

In the group that maintained their frequency of outdoor excursions, step counts significantly increased during midwinter compared with early winter (p < 0.01). In the group with a lower frequency of outdoor excursions, MVPA was significantly decreased during midwinter compared with early winter (p < 0.05). After adjusting for other variables, logistic regression analysis showed that weakness (odds ratio [OR]: 2.89, p < 0.05) was independently associated with a decline in step counts. Age (OR: 1.12, p < 0.05) and a change in frequency of outdoor excursions (OR: 0.75, p < 0.01) were independently associated with a decline in MVPA.

**Conclusions:**

Older persons should be supported in their attempts to go outdoors during midwinter. They should be provided with intervention programs to increase their physical activity at home.

## Background

Physical activity promotes well-being and physical and mental health, improves social connections and quality of life, and helps prevent disease. In addition, physical activity provides economic benefits and contributes to environmental sustainability [1]. Furthermore, non-communicable diseases account for approximately 60% of all deaths globally, and physical inactivity is one of the major risk factors that are associated with non-communicable diseases, causing an estimated 3.2 million deaths per year [2]. Therefore, increased physical activity is recommended for all age groups.

Unfortunately, physical activity decreases with age [3]. In older persons, a decline in physical activity is associated with poor physical performance [4], low bone mineral density [5], increased waist circumference and body mass index (BMI) [6], and elevated blood pressure and glucose levels [7]. Major environmental factors that affect physical activity are seasonal changes and climatic conditions, such as temperature, precipitation, and snow. Several studies have reported that the activity range for adults decreases during the winter season compared with that reported during the summer season [8-10]. Chan et al. also reported that adults’ step counts decreased by 3.6% for every 10 cm of snow on the ground [11].

With regard to the physical activity of older persons, Togo et al. demonstrated that the peak step counts among older Japanese adults occurred when the mean outdoor temperature was approximately 17°C in areas with little snow. When the temperature was above 17°C, physical activity decreased in a quadratic function, and this result was also observed at temperatures below 17°C. In addition, step counts decreased exponentially with increasing precipitation, and substantial changes were observed when the precipitation exceeded 10 mm [12]. However, few studies have examined changes in physical activity in older persons during the winter season, where heavy snowfall might cause a decrease in physical activity.

Therefore, this study aimed to examine changes in physical activity between early winter (little snow) and midwinter (heavy snowfall) in older persons who were living in northern Japan, and to identify the factors that might be associated with this change. We expect that these results will facilitate the development of a strategy to maintain the physical activity of older persons during the winter season.

## Methods

### Participants

This was a prospective study that was conducted in two phases (November 2012 and February 2013), as part of the Population-based and Inspiring Potential Activity for Old-old Inhabitants (PIPAOI) project. This study was performed in Bibai, Hokkaido, northern Japan. In 2012, Bibai had a population of approximately 25,000 persons, including 8,600 persons who were ≥65 years old. The details of the PIPAOI project have been described in detail previously [13,14].

The Ethics Committee of the Sapporo Medical University Hospital approved the present study’s protocol, and we conducted our study in accordance with the Declaration of Helsinki. Informed written consent was obtained from each participant prior to their enrolment.

A total of 1312 community-dwelling older persons (≥75 years old) were invited to participate in the study through an introductory letter. Of the 1312 persons who were invited to participate, 316 confirmed that they would attend a survey meeting, and 275 actually participated in the first round. Two hundred twenty-four individuals (81%) participated in the second round of this study. Participants were excluded from the analysis if they were hospitalised for >1 week in the 3 months before the study because of high blood pressure, stroke, cardiovascular disease, respiratory disease, diabetes, joint pain, or osteoporosis.

### Physical activity

The participants’ physical activity was monitored using the Kenz Lifecorder GS (Suzuken, Nagoya, Japan). This device was attached to the participant’s buttock. The data collection period was from when the participants got up in the morning to their falling asleep at night, for a 1-week period. Data were stored on a computer, and the mean daily step counts and moderate-vigorous physical activity (MVPA) times were calculated. When body movement data were not recorded for more than 2 consecutive hours, the data for that day were excluded [15]. Participants underwent measurements during early winter (November 2012) and midwinter (February 2013).

### Functional measures

The indicators of physical performance were handgrip strength and normal walking speed. The handgrip strength for a participant’s dominant hand was measured twice, and the highest result was used for analysis. The Smedley-type handheld dynamometer (Matsumiya Ika Seiki Mfg. Ltd., Tokyo, Japan) was used in the present study because handgrip strength is considered a valid indicator of general health status [16].

To assess normal walking speed, we used the mean of five test results. Participants were instructed to walk on a sheet of foot pressure sensors (Walk Way MW-1000; Anima, Tokyo, Japan), which are used to analyse walking performance [14]. Although the standard distance for this test is considered as 2.4 m, participants were asked to continue walking for an additional 2 m to ensure consistency in their pace throughout the task. For analysis, we used the data that were obtained between 2 m and 4.4 m from the start of the walkway.

We examined whether the participants included frail persons (weak and slow) using frailty criteria [17]. Weakness was defined as a grip strength of <29.0 kg (≤79 years old) or <23.5 kg (≥80 years old) in men, or as a grip strength of <17.5 kg (≤79 years old) or <12.5 kg (≥80 years old) in women. Slowness was defined as a walking speed of <73.0 m/min (≤79 years old) or <62.0 m/min (≥80 years old) in men, or as a walking speed of <67.0 m/min (≤79 years old) or <51.0 m/min (≥80 years old) in women [17].

### Questionnaire

For the present study, we used self-reported questionnaires to assess the participants’ functional capacity. The questionnaire addressed various factors, including the Tokyo Metropolitan Institute of Gerontology Index of Competence (TMIGIC), exercise habits, frequency of the outdoor excursions, length of outdoor excursions, snow shovelling, social support, and morbidity. Participants were asked to complete the questionnaires in advance, and we subsequently interviewed participants if portions of their questionnaire were not completed correctly.

We used the 13-item TMIGIC to evaluate high-level functional capacity of the participants. The validity and reliability of this index have been verified previously, and this index has been widely used in Japan as a tool to assess functional capacity [18]. Exercise habits were assessed according to the stages of change for exercise behaviour. We used a five-item Japanese questionnaire that was developed by Oka et al. [19], using stages that comprised “precontemplation”, “contemplation”, “preparation”, “action”, and “maintenance”. Participants were considered to have a habit of exercising if they indicated the stages of “action” or “maintenance” [14]. To estimate their mobility outside the home, participants were asked to indicate how often they went outdoors during 1 week (during early winter and midwinter), using an 8-point scale that ranged from 0 (less than once a week) to 7 (every day).

The length of outdoor excursions for early winter and midwinter was evaluated using the question “How long are you exposed to the sun?” Smoking behaviour was evaluated using the question “Do you currently smoke, or have you smoked in the past?” To evaluate habits of snow shovelling, participants were asked whether they shovelled snow in the winter. Social support was evaluated according to the number of support persons using the following statements: “I can consult this person when I am in trouble”, “I can consult this person when I feel physically sick”, “This person supports me in my daily life”, “This person will accompany me to the hospital if I am sick”, and “This person will care for me when I am bedridden” [20]. The morbidities that we evaluated were diabetes mellitus, hypertension, and hyperlipidaemia.

### Other covariates

For anthropometric measurements, participants were asked to wear light clothing without shoes. Height (to the nearest 0.1 cm) and body mass (to the nearest 0.1 kg) were recorded. The subject’s BMI was calculated using the standard formula: (weight [kg])/(height [m])^2^. Data regarding the climatic conditions in Bibai during the study period were obtained from the homepage of Japan’s Meteorological Agency [21], and these included temperature (average temperature >24 h,°C), precipitation (mm), and snowfall (cm).

### Statistical analysis

Data were analysed using SPSS software (version 20.0, SPSS Inc., Chicago, IL), with the significance level set at 5%. Differences between early winter and midwinter were assessed using the paired t-test and the Wilcoxon signed-rank test. Participants who went outdoors less during midwinter were defined as the declining group, and were compared with participants who maintained their frequency of outdoor excursions (maintenance group). The differences in physical activity between early winter and midwinter for each group were evaluated using the paired t-test. Logistic regression analysis was used to calculate the odds ratios (ORs) and 95% confidence intervals (95% CIs) for the effect of a 10% decline in step counts and MVPA during early winter and midwinter, while simultaneously controlling for potential confounding variables. The variables that were considered in the models were age, sex, BMI, weakness, slowness, exercise habits, smoking, TMIGIC, social support, morbidity, snow-shovelling habits, and a change in frequency (between early winter and midwinter) of outdoor excursions. Logistic regression analysis was performed using univariate analysis (Model 1) and multivariate analysis (Model 2), which was adjusted for all of the confounding variables.

## Results

### Changes in physical activity according to season

**Figure 1:**
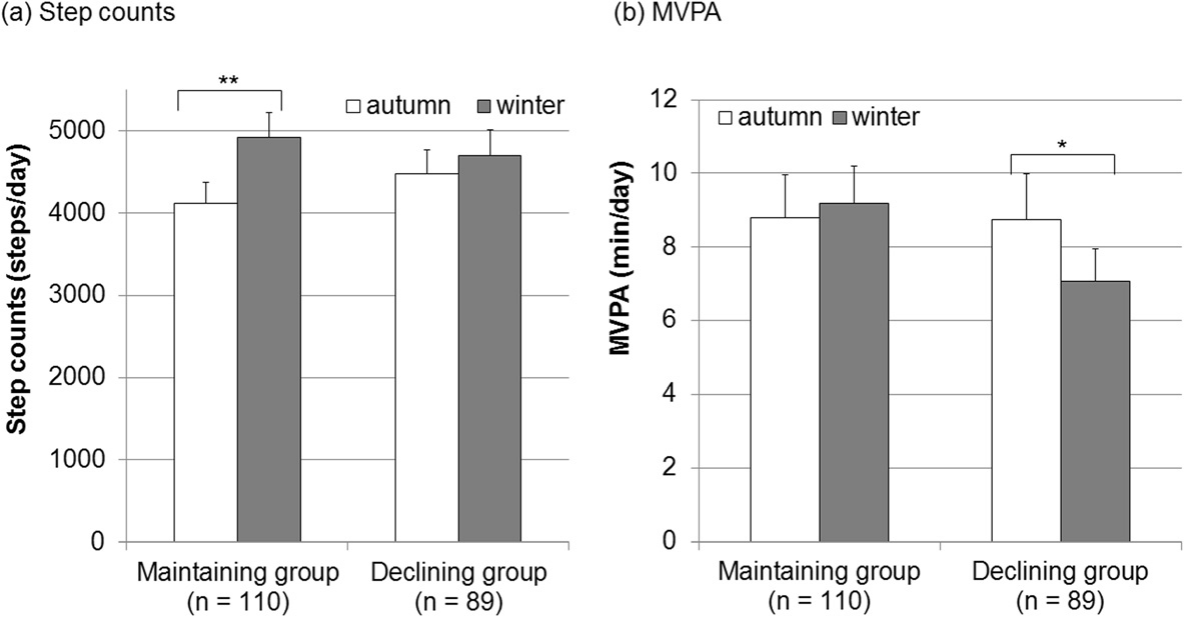
**Differences in physical activity between early winter and midwinter in the maintenance and declining groups. (a)** Step counts, **(b)** MVPA. MVPA: moderate-vigorous physical activity. *p < 0.05, **p < 0.01; bars indicate standard errors.

Table 1 shows the characteristics of the 201 eligible participants (83 men and 118 women; mean age: 79.7 ± 3.8 years). Step counts were significantly higher during midwinter than during early winter (p < 0.01). However, MVPA did not significantly change between the two periods. The frequency of outdoor excursions and the length of exposure to sunlight were significantly lower during midwinter than during early winter (p < 0.01, Table 2). After dividing the participants into the maintenance and declining groups, based on the frequency of their outdoor excursions, step counts were found to significantly increase during midwinter compared with early winter in the maintenance group (p < 0.01). In addition, MVPA was significantly decreased during midwinter compared with during early winter in the declining group (p < 0.05, Figure 1).

**Table 1:** **Characteristics of the study participants (n = 201)**

Age (years)^a)^	79.7 ± 3.8
Sex (female)^c)^	118 (58.7)
BMI (kg/m^2^)^a)^	24.1 ± 3.2
Weakness (yes)^c)^	23 (11.4)
Slowness (yes)^c)^	43 (21.4)
Exercise habit (yes)^c)^	86 (42.8)
Smoking (yes)^c)^	64 (31.8)
TMIGIC (point)^b)^	13 [11, 13]
Social support score (point)^b)^	5 [4, 5]
Morbidity (yes)^c)^	150 (74.6)
Snow shovelling habit (yes)^c)^	165 (82.1)

BMI: body mass index.

TMIGIC: Tokyo Metropolitan Institute of Gerontology index of competence.

^a)^Mean ± standard deviation.

^b)^Median [interquartile range].

^c)^Number (%).

**Table 2:** **Differences in outdoor excursions and physical activity according to season (n = 201)**

	**Early winter (November)**	**Midwinter (February)**
Weather condition		
Temperature (monthly average,°C)	3.5	−7.2
Precipitation (monthly average, mm)	2.8	4.7
Snowfall (monthly average, cm)	2.4	6.8
(monthly peak, cm)	19	146
Step counts (steps/day)^a)^	4255.0 ± 2683.3	4809.8 ± 3116.3^**^
MVPA (min/day)^a)^	8.7 ± 12.1	8.2 ± 9.6
Frequency of outdoor excursions (days/week)^b)^	4 [3, 6]	3 [2, 5]^**^
A change in frequency of outdoor excursions (midwinter ― early winter)	0 [−1, 0]	
Length of outdoor excursions (min/day)^a)^	95.3 ± 94.8	74.0 ± 69.0^**^

MVPA: moderate-vigorous physical activity.

^a)^Mean ± standard deviation. The paired t-test was used to compare continuous variables between early winter and midwinter.

^b)^Median [interquartile range]. The Wilcoxon signed-rank test was used to compare ordinal variables between early winter and midwinter.

^**^p < 0.01.

### Factors associated with a decline in physical activity

The results of logistic regression analysis are shown in Table 3. Univariate logistic regression analysis (Model 1) showed that weakness was associated with a decline in step counts (OR: 2.74, 95% CI: 1.13–6.62, p < 0.05). Multivariate logistic analysis (Model 2) also showed that weakness was independently associated with a decline in step counts (OR: 2.89, 95% CI: 1.102–7.58, p < 0.05). In Model 1, age (OR: 1.08, 95% CI: 1.00–1.16, p < 0.05) and a change in frequency of outdoor excursions (OR: 0.81, 95% CI: 0.67–0.97, p < 0.05) were associated with a decline in MVPA. In Model 2, age (OR: 1.12, 95% CI: 1.03–1.22, p < 0.05) and a change in frequency of outdoor excursions (OR: 0.75, 95% CI: 0.62–0.92, p < 0.01) were also independently associated with a decline in MVPA.

**Table 3:** **Logistic regression model of the factors that were associated with a change in physical activity**

	**Step counts**				**MVPA**			
	**Decline (n = 64) vs. Other (n = 137)**				**Decline (n = 89) vs. Other (n = 112)**			
	**Model 1**		**Model 2**		**Model 1**		**Model 2**	
**Risk factor**	***OR***	***95% CI***	***OR***	***95% CI***	***OR***	***95% CI***	***OR***	***95% CI***
Age (years)	1.05	0.97 ― 1.13	1.07	0.98 ― 1.17	1.08^*^	1.00 ― 1.16	1.12^*^	1.03 ― 1.22
Sex (male: 1, female: 2)	1.15	0.62 ― 2.10	0.98	0.39 ― 2.43	0.90	0.51 ― 1.59	1.02	0.44 ― 2.40
BMI (kg/m^2^)	1.05	0.96 ― 1.15	1.04	0.94 ― 1.16	1.00	0.92 ― 1.09	1.02	0.92 ― 1.13
Weakness (yes: 1, no: 0)	2.74^*^	1.13 ― 6.62	2.89^*^	1.10 ― 7.58	1.48	0.62 ― 3.54	1.86	0.71 ― 4.89
Slowness (yes: 1, no: 0)	1.79	0.89 ― 3.60	1.72	0.77 ― 3.86	0.89	0.45 ― 1.77	0.74	0.33 ― 1.64
Exercise habit (yes: 1, no: 0)	1.13	0.62 ― 2.06	1.31	0.66 ― 2.62	1.14	0.65 ― 2.00	1.37	0.72 ― 2.61
Smoking (yes: 1, no: 0)	0.82	0.43 ― 1.58	0.87	0.35 ― 2.14	1.29	0.71 ― 2.35	1.40	0.60 ― 3.25
TMIGIC (score)	1.04	0.89 ― 1.20	1.07	0.88 ― 1.30	1.02	0.89 ― 1.17	1.07	0.90 ― 1.28
Social support (score)	1.13	0.89 ― 1.43	1.09	0.83 ― 1.43	0.95	0.77 ― 1.17	0.90	0.71 ― 1.14
Morbidity (yes: 1, no: 0)	1.32	0.65 ― 2.67	1.33	0.61 ― 2.89	1.65	0.85 ― 3.18	1.95	0.93 ― 4.08
Snow shovelling (yes: 1, no: 0)	0.68	0.32 ― 1.44	0.81	0.33 ― 2.01	0.99	0.48 ― 2.05	1.02	0.42 ― 2.49
A change in the frequency of outdoor excursions (midwinter ― early winter, days)	0.88	0.73 ― 1.06	0.83	0.68 ― 1.02	0.81^*^	0.67 ― 0.97	0.75^**^	0.62 ― 0.92

MVPA: moderate-vigorous physical activity.

BMI: body mass index.

TMIGIC: Tokyo Metropolitan Institute of Gerontology index of competence.

The dependent variable was considered a decline of 10% during early winter and midwinter of each physical activity (decline = 1, other = 0).

Model 1: univariate model.

Model 2: Multivariate model that was adjusted for age, sex, BMI, weakness, slowness, exercise habits, smoking, TMIGIC, social support, morbidity, snow-shovelling habits, and a change in frequency of outdoor excursions.

^*^p < 0.05, ^**^ p < 0.01.

## Discussion

The present study examined changes in physical activity that were observed between early winter and midwinter in older persons who were living in a northern area of Japan, and evaluated the factors that were associated with this change. We found a significant increase in step counts in older subjects during midwinter compared with early winter. In contrast, the frequency and length of outdoor excursions significantly decreased over the winter season.

While the maintenance group (no decrease in frequency of outdoor excursions during midwinter) had higher step counts during midwinter compared with early winter, the declining group (reduction in the frequency of outdoor excursions during midwinter) maintained their step counts. In addition, logistic regression showed that only weakness was independently associated with a decline in step counts. These results suggest that a decline in the frequency of outdoor excursions does not affect the decline in step counts. Our findings also indicated that the declining group maintained their step counts through household activities. In addition, our results indicated that weakness, as measured using an index of muscle weakness, affected the decline in step counts. Previous studies have reported similar results, where decreased muscle strength was associated with decreased physical activity [22,23]. Furthermore, grip strength and knee extension strength affect physical activity through motor disabilities [24], which is consistent with the findings of our study.

In our study, the maintenance group had similar MVPA during early winter and midwinter, although MVPA was significantly decreased during midwinter compared with early winter in the declining group. This result suggested that the participants who performed indoor activity used low intensity activities, which caused a decline in their MVPA. Interestingly, our logistic regression model showed that a change in the frequency of outdoor excursions affected the decline in MVPA from early winter to midwinter, even after adjusting for various confounding factors. Tanaka et al. reported that the frequency of outdoor excursions was independently associated with physical activity levels in elderly Japanese community-dwelling people [25]. Various factors might affect a decline in the frequency of outdoor excursions during midwinter, including cold temperatures, snow, slippery roads, and a high risk of falling. Similarly, a previous study showed that cold temperatures and snow affected physical activity during winter [12]. This previous study demonstrated that the greatest number of steps that were taken by older Japanese adults coincided with a mean outdoor temperature of approximately 17°C; physical activity decreased in a quadratic function when temperatures were markedly above or below 17°C. Similarly, Yasunaga et al. reported a decline in physical activity during the winter season, using accelerometer data that were collected throughout the year [3]. Furthermore, step counts are known to decrease by 3.6% (range, 0.8–6.4%) for every 10 cm of accumulation of snow, after adjusting for sex and BMI [11]. However, the relationship between step counts and the frequency of outdoor excursions and that between MVPA and the frequency of outdoor excursions were different in this study. Step counts might be maintained through household activities, although MVPA cannot be maintained exclusively by household activities. Therefore, to maintain MVPA and promote better cardiorespiratory function, muscular fitness, and bone health, we suggest that the frequency of outdoor excursions should be maintained in elderly people during the midwinter season.

Although a previous study reported that physical activity decreases during winter [8-10], the present study showed that step counts increased and MVPA was maintained during midwinter for all of the participants. The older subjects who participated in this study were relatively healthy, as confirmed by a high TMIGIC score, which was used as an index of the instrumental activities of daily living in a previous study [18]. In addition, a previous study reported an association between TMIGIC scores and physical activity levels [25], and most of the participants had a high social support score. Furthermore, Ishii et al. analysed the factors that affect physical activity, using structural equation modelling in Japanese adults (20–79 years old), and reported that social support affected physical activity through self-efficacy [26]. Our results indicated that step counts significantly increased, while MVPA was maintained, between early winter and midwinter.

We conclude that a change in the frequency of outdoor excursions affected the participants’ physical activity levels. Our results highlight the importance of making outdoor excursions and increasing physical activity in the home during midwinter. Chan and Ryan reported that maintaining physical activity during winter is essential for maintaining health, and emphasised the need for proper protective equipment and footwear when participating in winter activities, such as skating, snowshoeing, and cross-country skiing [27]. In addition, Tucker and Gilliland suggested that providing opportunities for indoor activities during the cold and wet seasons helps to foster regular physical activity throughout the year [28].

Although the habit of snow shovelling was not independently associated with step counts or MVPA, many people who shovelled snow (82% of participants) were included in this study. However, the details regarding snow shovelling, such as duration, frequency, and intensity, were unclear, and these factors may have affected the participants’ physical activity levels.

A limitation of the present study was the physical condition of the participants. Although we examined participants who were living in the Bibai area during late November, snow had already fallen at that time. Therefore, the participants’ physical activity levels might have declined before the examination. Furthermore, the current study examined the differences in physical activity levels between early winter and midwinter. Therefore, a distinct seasonal change was not observed during the study period. In addition, the present study included older subjects with a high functional capacity, which limits the generalization of our results to frail and disabled older persons. Furthermore, this study did not identify the causality of the relationship between physical activity and the frequency of winter outdoor excursions. To elucidate this causality, a randomised control trial is required to examine if an intervention could enhance the frequency of midwinter outdoor excursions.

## Conclusions

The present study examined the changes in physical activity that were observed during midwinter in older persons who were living in northern Japan, and evaluated the factors that were associated with these changes. We found that MVPA was significantly decreased in persons who went outdoors less during midwinter, although their step counts were maintained in that period. Muscle weakness, aging, and the frequency of outdoor excursions are all important factors that affect a decline in physical activity during midwinter. Therefore, older persons should be supported in going outdoors during midwinter, and they should be provided with intervention programs that increase their physical activity at home.
